# Long noncoding RNA expression in peripheral blood mononuclear cells and suicide risk in Chinese patients with major depressive disorder

**DOI:** 10.1002/brb3.711

**Published:** 2017-05-02

**Authors:** Xuelian Cui, Wei Niu, Lingming Kong, Mingjun He, Kunhong Jiang, Shengdong Chen, Aifang Zhong, Wanshuai Li, Jim Lu, Liyi Zhang

**Affiliations:** ^1^ Health Care DepartmentChangzhou Maternity and Child Health Care Hospital Affiliated with Nanjing Medical UniversityChangzhouChina; ^2^ Department of RehabilitationNo. 102 Hospital of Chinese People's Liberation ArmyChangzhouChina; ^3^ Prevention and Treatment Center for Psychological DiseasesNo.102 Hospital of Chinese People's Liberation ArmyChangzhouChina; ^4^ Department of NeurologyNo.102 Hospital of Chinese People's Liberation ArmyChangzhouChina; ^5^ Department of LaboratoryNo.102 Hospital of Chinese People's Liberation ArmyChangzhouChina; ^6^ Gopath Diagnostic Laboratory Co. LtdChangzhouChina; ^7^ Gopath Laboratories LLCBuffalo GroveILUSA

**Keywords:** LncRNA, major depressive disorder, prediction equation, suicide risk

## Abstract

**Background:**

WHO stated that nearly one million people commit suicide every year worldly, and 40% of the suicide completer suffered from depression. The primary aim of this study was to explore the association between long noncoding RNAs (lncRNAs) expression in peripheral blood mononuclear cells (PBMCs) and suicide risk of patients with major depressive disorder (MDD).

**Methods:**

Using Human LncRNA 3.0 microarray profiling which includes 30,586 human lncRNAs and RT‐PCR, six down‐regulated lncRNAs were identified differentially expressed in MDD patients. According to suicidal ideation and suicidal attempt, the suicide risk of MDD patients was classified into suicidal ideation versus no suicidal ideation groups, and past attempt versus no past attempt groups, respectively. The expression of six lncRNAs in MDD patients and controls were examined by RT‐PCR.

**Results:**

The expression of six lncRNAs had significant differences between no suicidal ideation, suicidal ideation, and controls; corresponding lncRNAs associated with suicidal attempt had remarkable differences between no past attempt, past attempt, and controls. Additionally, only the expression of lncRNAs in suicidal ideation group and past attempt group markedly declined compared with controls.

**Conclusions:**

This study indicated that the expression of six down‐regulated lncRNAs had a negative association with suicide risk in MDD patients, and the expression of lncRNAs in PBMCs could have the potential to help clinician judge the suicide risk of MDD patients to provide timely treatment and prevent suicide.

## 1 Introduction

Suicide is a complex public health concern and nearly one million people die of suicide every year (WHO 2012). Suicidal behavior includes a broad spectrum, for example, suicide completer, highly lethal suicide attempts, and low deadly suicide attempts (Mann, 2002). The unipolar depression and depression phase for bipolar disorder are the highest risk of suicide, 15–20% of the depressed patients ended the life with suicide (Goodwin et al., 2003). More than half of the depressed patients who died of suicide had seen their family doctor within three months prior to the implementation of suicide (Pirkis & Burgess, 1998). These data highlight the importance of predicting suicide risk accurately, especially in general hospital and psychiatric emergency services (WHO 2012). To date, in order to assess a patient's immediate suicide risk, a lot of information are needed which may be collected during the clinical interview, including risk and protective factors, such as early‐life trauma, stressful life events, impulsive aggressive traits, psychopathology, personal and family history, identification models, data on physical health, and social support network (Bertolote, Mello‐Santos, & Botega, 2010; Mann et al., 2009). Paradoxically, many GPs have received no formal training in suicide assessment (Bajaj et al., 2008). Suicide risk assessment is challenging for several reasons. Conventional approaches to risk assessment, for example, suicidal risk scales (Linehan, Comtois, Brown, Heard, & Wagner, 2006; Nock, Holmberg, Photos, & Michel, 2007; Reilly‐Harrington et al., 2016) rely on patient's self‐reporting, but individuals who are truly suicidal often do not share that information. Besides, none of the suicidal risk scales have shown reliable efficiency in determining the suicidal risk, and few have been tested on their predictive ability for suicidal behavior (Roos, Sareen, & Bolton, 2013). An important shift in the science underlying risk assessment scales has been the move from interview‐dependent tools to interview independent tools. One strategy proposed by the National Action Alliance for Suicide Prevention to predict suicide is requiring the identification of reliable biomarkers capable of identifying those at current or future risk (NAAfSPRPT 2014). Peripheral biomarkers for suicide have been performed, such as genetic polymorphisms of 5‐HT (de Medeiros Alves, Bezerra, de Andrade, de Melo Neto, & Nardi, 2015; Pandey & Dwivedi, 2006), NE function and its metabolite 3‐methoxy‐4‐hydroxyphenylglycol (MHPG) (Sher et al., 2006; Tripodianakis, Markianos, Sarantidis, & Agouridaki, 2002), abnormal HPA axis (Yerevanian, Feusner, Koek, & Mintz, 2005), and low plasma BDNF (Deveci, Aydemir, Taskin, Taneli, & Esen‐Danaci, 2007; Pandey et al., 2010).

With the development of RNA deep sequencing technology and bioinformatics, recent work has revealed that early‐life adversity can mediate suicide risk through long‐term epigenetic regulation of gene expression (Turecki & Brent, 2015). Noncoding RNAs (ncRNAs) are identified as potential biomarkers with capacity of predicting suicidal behavior from blood. MicroRNA‐301a in the cortex of depressed suicide completers represents a promising biomarker for detecting suicidal behaviors (Guintivano et al., 2014; Smalheiser et al., 2012). Long noncoding RNA (LncRNA), one type of ncRNA, is more than 200nt in length, not encoding proteins itself, but regulating gene expression in multi‐level form of RNA, such as epigenetic regulation, transcriptional regulation, and posttranscription regulation. The expression of an antisense lncRNA was high in postmortem brain of violent suicide completers, but not in depressed patients (Punzi et al., 2014).

Using Human LncRNA 3.0 microarray profiling and RT‐PCR in more than 100 samples, six down‐regulated lncRNAs (TCONS_00019174, ENST00000566208, NONHSAG045500, ENST00000517573, NONHSAT034045, and NONHSAT142707) have been identified as potential diagnostic and therapeutic biomarkers for MDD (Cui et al., 2016). In this study, we tried to further explore the association between lncRNAs expression and suicide risk in MDD, to predict and prevent the suicidal behavior of depressed patients earlier.

## 2 Materials and methods

### 2.1 Subjects

A total of 120 patients who met the criteria of MDD as described in the Diagnostic and Statistical Manual of Mental Disorders, 4th Edition (DSM‐IV) were enrolled from Changzhou Maternity and Child Health Care Hospital and No.102 Hospital of the Chinese People's Liberation Army from May 2014 to February 2015. Diagnoses were independently made by two mental physician using the Chinese version of the modified Structured Clinical Interview for DSM‐IV, patient version (SCID‐I/P) (First, Spitzer, Gibbon, Williams, & Davies, 1995), the inter‐rater reliability between two psychiatrists was 0.87, and all patients were assessed using the 24‐item Hamilton Rating Scale (HAMD_24_)(Hamilton, 1967). The inclusion criteria were as follows: (1) patients were either maiden visitors or precedent for any clinical treatment, (2) patients had not taken any antidepressants for at least 3 months before enrollment in the study, (3) the age of patients ranged between 18 and 60 years old, (4) patients had no previous history of organic disease (such as heart disease, diabetes, or Parkinson's disease), (5) female patients not currently pregnant, (6) patients had no severe negative life events within the last 6 months before their diagnosis, (7) patients had no other psychiatric disorders.

Totally, 63 normal controls were recruited from the community nearby, having no family history of major psychiatric disorders (schizophrenia, bipolar disorder, MMD, and psychoactive substance use disorder) and no history of severe traumatic events within 6 months of being enrolled in the study. Normal controls were also assessed using HAMD_24_ and the modified SCID–I/P to rule out prior incidence of mental disease and suicide‐related thoughts or behaviors. Patients and controls were matched in gender, age, marital status, and ethnicity at a ratio of 2:1. The study was approved by the Ethical Committee for Medicine of Changzhou Maternity and Child Health Care Hospital and No.102 Hospital of Chinese People's Liberation Army. All participants and their legal guardians gave written informed consent.

### 2.2 RT‐ PCR

Whole blood (5 ml) samples from 120 MDD patients and 63 controls were collected to validate the six down‐regulated lncRNAs (TCONS_00019174, ENST00000566208, NONHSAG045500, ENST00000517573, NONHSAT034045, and NONHSAT142707) expressions using RT‐PCR.

Total RNAs were extracted from the PBMCs using TRIzol reagent (Invitrogen^®^, USA) for quantitative detection of lncRNAs. Complementary DNA (cDNA) was synthesized using the TaqMan RNA Reverse Transcription Kit (ABI., USA) according to the manufacturer's protocol. Each RT reaction included 10 μl of total RNA, 3.0 μl TaqMan MicroRNA Assay, 4.16 μl Nuclease‐free water, 0.19 μl Rnase Inhibitor, 1 μl Multiscribe Reverse Transcriptase, 0.15 μl dNTP, 1.5 μl 10 × RT Buffer, in a total volume of 15 μl. Reactions were implemented as per the following conditions: 30 min at 16°C, 30 min at 42°C, 5 min at 85°C, and 10 min at 4°C. Real‐time PCR was performed using the Applied Biosystems 7900HT Real‐Time PCR System (Applied Biosystems, Inc., USA). Each sample was assayed in duplicate. The SDS 2.3 software (Applied Biosystems, Inc.) and DataAssist v3.0 software were used to collect the data. After normalization to β‐Actin, the expression levels of lncRNAs were calculated using the 2^−ΔΔCt^ method.

### 2.3 Suicide risk assessment

According to whether patients had the suicidal ideation or suicidal attempt, MDD patients were classified into no suicidal ideation group and suicidal ideation group, no past attempt group and past attempt group, respectively.

### 2.4 Statistical analysis

All statistical analyses were carried out using DataAssist version 3.0 software, SPSS version 20.0 software (Chicago, IL, USA), and Graphpad Prism 5 (Graphad Software Inc., San Diego, CA, USA). Demographic variables were compared with a chi‐square test for qualitative variables and t‐test for quantitative variables. The difference in lncRNAs between each suicide risk group and controls were analyzed by one‐way ANOVA. *p* values of <.05 (two‐tailed) were considered statistically significant.

## 3 Results

### 3.1 Demographic data of the MDD patients and control group

Using chi‐square and t‐test, there were no significant differences between the suicide risk groups and controls with regard to age, gender, ethnicity, and marital status, but the HAMD scores were significantly different (Table 1).

**Table 1:** Demographic variables of the MDD patients and controls

	MDD (*n *= 120)	Controls (*n *= 63)	*p* value
Sex			
Male	47 (39.2%)	30 (47.6%)	.478
Female	73 (60.8%)	33 (52.4%)	
Age, years			
Mean	36.41 (16.6)	39.58 (13.4)	.254
Range	18–60	20–55	
Ethnicity			
Han	120	63	
Ethnic minority	0	0	
Marital status			
Married	88 (73.3%)	45 (71.4%)	.75
Unmarried	32 (26.7%)	18 (28.6%)	
HAMD score			
Mean	27.2 (7.19)	7.5 (2.3)	<.01

MDD, major depressive disorder; HAMD, Hamilton depression scale.

### 3.2 RT‐PCR

Six down‐regulated lncRNAs (TCONS_00019174, ENST00000566208, NONHSAG045500, ENST00000517573, NONHSAT034045, and NONHSAT142707) were validated in 120 MDD patients and 63 healthy controls using RT‐PCR, the results and six lncRNAs location are shown in Table 2.

**Table 2:** Comparison of six lncRNAs expression between MDD patients and controls ($\bar{x}$ ±s)

Probes	chr	MDD (*n *= 120)	NC (*n *= 63)	*p* value
TCONS_00019174	chr11:116367616‐116371347	6.95 ± 2.46	5.77 ± 2.78	.0153
ENST00000566208	chr16:8348494‐8349774	6.36 ± 2.51	5.34 ± 2.70	.0373
NONHSAG045500	chr6:170426388‐170430482	8.24 ± 2.52	7.23 ± 2.57	.0376
ENST00000517573	chr8:20831308‐20832540	7.50 ± 2.69	6.34 ± 2.69	.0237
NONHSAT034045	chr13:55976660‐55981315	7.01 ± 2.71	5.98 ± 2.76	.0457
NONHSAT142707	chr16:56716393‐56718108	9.14 ± 2.57	7.97 ± 2.76	.0193

MDD, major depressive disorder; NC, normal control.

### 3.3 Comparison of lncRNAs expression between no suicidal ideation group, suicidal ideation group, and control group

Using ANOVA, the expression of six down‐regulated lncRNAs had significant difference between no suicidal ideation group, suicidal ideation group, and controls (*F *= 7.93–11.11, *p *< .01). The expression in suicidal ideation group was significantly lower than other two groups, as shown in Table 3.

**Table 3:** Comparison of lncRNAs expression between suicidal ideation groups and controls ($\bar{x}$ ±s)

Probes	No suicidal ideation (*n *= 63)	Suicidal ideation (*n *= 57)	Controls (*n *= 43)	*F*	*p*
TCONS_00019174	6.93 ± 2.28	4.75 ± 3.68^a^	6.40 ± 3.18	7.93	<.01
ENST00000566208	6.34 ± 2.32	4.14 ± 3.69^a^	5.97 ± 3.13	8.47	<.01
NONHSAG045500	8.27 ± 2.29	5.71 ± 3.89^a^	5.97 ± 3.13	11.11	<.01
ENST00000517573	7.57 ± 2.48	5.01 ± 3.89^a^	7.02 ± 3.19	10.08	<.01
NONHSAT034045	7.00 ± 2.50	4.46 ± 4.09^a^	6.66 ± 3.25	9.78	<.01
NONHSAT142707	9.20 ± 2.34	6.81 ± 3.84^a^	8.65 ± 3.26	9.01	<.01

a There were significant difference between suicidal ideation group and no suicidal ideation group (*p* < .01), suicidal ideation group and controls (*p* < .01). No significant difference existed between no suicidal ideation group and controls (*p* > .05).

### 3.4 Comparison of lncRNAs expression between no past attempt group, past attempt group, and control group

**Figure 1:**
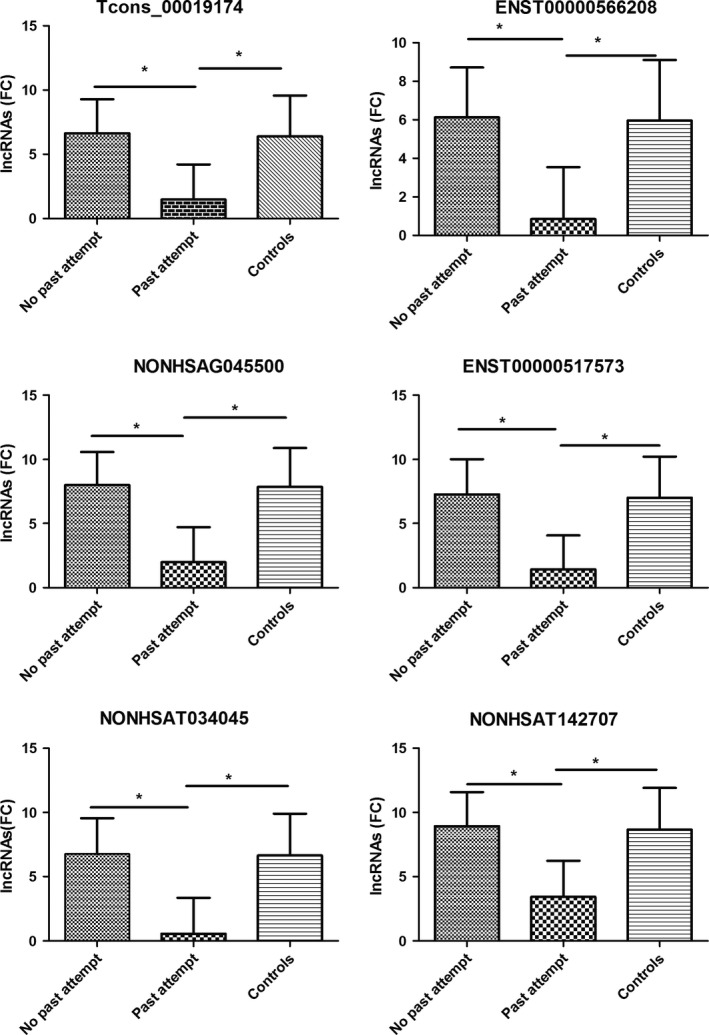
Comparison of lncRNAs expression between attempt groups and controls. *p<0.05

By means of ANOVA, the expression of six down‐regulated lncRNAs had significant difference between no past attempt group (*n *= 101), past attempt group, (*n *= 19) and controls (*n *= 63) (*F *= 30.1–40.8, *p* < .01). The expression in past attempt group was significantly lower than other two groups (Figure 1).

## 4 Discussion

Suicide is a complex behavior involving not only genetics and environment but also gene—environment interactions. Evidence based on clinicians’ subjective observation and inquest, the case history (including the suicidal ideation and behavior as well as the patient's nonverbal communication style) led to misdiagnosis and missed diagnosis easily (Deisenhammer et al., 2004). Mental health clinicians with different ethnicity (Sohler & Bromet, 2003), gender (Crosby & Sprock, 2004), and age (James & Haley, 1995) had significant heterogeneity in estimating suicide risk, and rarely predict suicide at a rate greater than chance (Garb, 2005). Thus, a quantifiable indicator concerning the molecular and cellular mechanisms underlying major depression and suicidal behavior is urgently needed to be explored. Candidate genes have been investigated in the postmortem brains of suicide victims, such as Gamma‐amino butyric acid type A (GABA_A_) receptor (Poulter et al., 2008), Glucocorticoid receptor (hGR1 h) (Labonte et al., 2012), and brain‐derived neurotrophic factor (BDNF) promoter (Keller et al., 2010), but there is common genetic predisposition between SZ and MDD (Chen et al., 2015; Eker, Yavasci, Cangur, Kirli, & Sarandol, 2014; He et al., 2014). Therefore, Genome‐wide association studies (GWAS) have been inconsistent in elucidating the association between genes and suicidal behavior, and make the heritability of suicidal behavior still unclear (Bani‐Fatemi, Howe, & Luca, 2015). The interaction of genetics and environment has given rise to the potential role of epigenetics in suicidal behavior (Mann & Currier, 2010). Epigenetic theory can explain how current candidate genes confer risk for suicidal behavior, and the heritability of these risks beyond the variation present in DNA static mode. Several epigenetic mechanisms (e.g., DNA methylation, histone modification, RNA interference), as potential epigenetic markers of gene alteration in suicidal behavior have been elucidated. There is a significant association between functional single‐nucleotide polymorphisms (SNPs) in epigenetic regulatory genes, DNA methyltransferases (DNMT1 and DNMT3b), and suicide attempts in psychiatric patients (Murphy et al., 2013). The increase in H3K27 (Histone 3 Lysine 27) methylation has a significant correlation with the decreased TrkB.T1 (Tropomyosin‐related kinase B) expression in the orbitofrontal cortex of suicide completers (Ernst, Chen, & Turecki, 2009). A significant increase in hsa_miR_185 expression was associated with low TrkB.T1 cortical expression in suicide victims (Maussion et al., 2012).

Previous studies about suicidal behavior were based on the postmortem brain of suicide completers. In our view, choosing brain tissue of dead people as research materials has two limitations. Firstly, it is hard and unethical to get brain tissue when individual is still alive; secondly, suicide completer can never be traced back to one single cause, it is meaningless to know the suicide risk for dead individuals, only what we need to know is the patients’ power of suicide ideation and possibility of committing suicide behavior when they are still alive. Thus, in this study, we chose PBMCs in patients’ venous blood as sample and differentially expressed lncRNAs in MDD patients alive as object to determine whether the expression of lncRNAs in PBMCs had significant difference between patients with suicide ideation and without suicide ideation, with past suicide attempt and without past suicide attempt. The results demonstrated that patients with suicide ideation had significantly lower lncRNAs expression than those without suicide ideation and controls. Similarly, the expression of six lncRNAs markedly declined in patients who had already attempted suicide compared with those without past attempt and controls. Epigenetic modifications are reversible, and in a sense this modification is a dynamic process, allowing the cell to become more responsive to environment stimuli (Lorincz, Dickerson, Schmitt, & Groudine, 2004). In previous studies, we observed that the expression of lncRNAs in MDD patients changed following the antidepressant treatment, even returned to normal levels when depressive symptoms were relieved (Song et al., 2014; Zhang et al., 2014). Therefore, we concluded that these six lncRNAs is reversible and have dynamic process in the development of MDD. lncRNA have been shown to be widely involved in various biological processes in the central nervous system, such as hippocampal development, oligodendrocyte myelination, brain aging, post‐CREB (cAMP response element binding protein) and PGC1‐alpha transcriptional regulation, GABA neurons, and G protein‐coupled receptor signal transduction pathways (Ponjavic, Oliver, Lunter, & Ponting, 2009). By Gene Ontology analysis, the lncRNAs in this study were mainly involved in the following functions: (1) protein transport, (2) translational elongation, (3) protein complex biogenesis, (4) establishment of protein localization, (5) protein complex assembly, and (6) translation. Furthermore, the results of Kyoto Encyclopedia of Genes and Genomes (KEGG) analysis showed that these lncRNAs had close associations with (1) Alzheimer's disease, (2) Huntington's disease, and (3) Parkinson's disease. These functions suggested that six lncRNAs play a critical role in the onset and development of nervous diseases; they could be objective indicators which dynamically reflect the health status of central nervous system, including the suicidal ideation and suicidal attempt in MDD patients.

In conclusion, suicide is a complex behavior, candidate suicide genes done by GWAS are inconsistent in elucidating the association between genes and suicidal behavior, but lncRNAs, as continuous and quantified indicators, could be used to help judge the suicide risk of MDD patients and make preventive action.

## Conflict of Interest

None declared.
